# Assessing the Understanding of Pharmaceutical Pictograms among Cultural Minorities: The Example of Hindu Individuals Communicating in European Portuguese

**DOI:** 10.3390/pharmacy6010022

**Published:** 2018-03-05

**Authors:** Lakhan Kanji, Sensen Xu, Afonso Cavaco

**Affiliations:** Department of Social Pharmacy, Faculty of Pharmacy, University of Lisbon. Av. Prof. Gama Pinto, 1649-003 Lisboa, Portugal; lakhan@hotmail.com (L.K.); sensen.58@hotmail.com (S.X.)

**Keywords:** pharmaceutical pictograms, written health communication, Hindu community, USP, FIP PictoRx, Portugal

## Abstract

One of the sources of poor health outcomes is the lack of compliance with the prescribed treatment plans, often due to communication barriers between healthcare professionals and patients. Pictograms are a form of communication that conveys meaning through its pictorial resemblance to a physical object or an action. Pharmaceutical pictograms are often associated with a better comprehension of treatment regimens, although their use is still subject to limitations. The main goal of this study was to examine the potential understanding of pharmaceutical pictograms by a cultural minority when providing patient information while comparing the effectiveness of two reference systems (United States Pharmacopeia USP and International Pharmacy Federation FIP) for this purpose. A self-administered questionnaire was developed comprising 30 pictograms, 15 selected from the United States Pharmacopeia Dispensing Information and the equivalent from the International Pharmaceutical Federation. The questionnaire comprised plain instructions, socio-demographic data, self-reported language fluency and pictogram labels in Portuguese presented to conveniently selected members of the Hindu community of Lisbon (Portugal) until reaching a quota of 50. Participants showed difficulties in understanding some pictograms, which was related to the self-reported reduced fluency in Portuguese. Overall, the interpretation of USP pictograms was better than FIP ones, as well as for pictograms composed of multiple images, presenting a negative reading, or when conveying information unrelated to medication instructions. Even using internationally validated pictograms, added care should be taken when community pharmacists use such communication resources with cultural minorities. It is important not to disregard other forms of patient communication and information, considering pictograms as a complement to other forms of patient counselling.

## 1. Introduction

It is well accepted that it is the responsibility of community pharmacists to actively contribute to the safe and effective use of medications [1,2]. While their primary mission is to assure the quality of the products dispensed, the current focus on pharmaceutical care practice adds a professional responsibility towards patient medication outcomes [3]. Community pharmacists are actively contributing to improving medication usage, including medication compliance, treatment effectiveness and adverse events monitoring [1,3]. Medication compliance can be defined as the extent to which a patient acts in accordance with the prescribed dosing regimen [4]. Inadequate compliance has important negative patient outcomes [5,6] and usually emerges from therapy costs and complexity of the regimen, being communication barriers between health professionals and patients also known to contribute to non-compliance [6].

Communication barriers may arise from speech or hearing impairments but are commonly a consequence of language issues related to schooling or literacy limitations [7]. Lack of therapy compliance is frequent for the elderly and those who do not speak the same language as the healthcare providers [8,9,10,11]. Despite efforts to implement Esperanto or basic English, communication issues persist based on language differences between communities including alphabet, lexical, syntactic and semantic variations, even between bordering counties [12]. To overcome communication issues, information can be conveyed using pictures, symbols, audiotapes or interpreters [9]. One widely known resource, frequently considered a beneficial solution, is the use of pictograms.

### 1.1. What are Pictograms?

Pictograms are graphic representations of objects or actions conveying a meaning which should be independent of any particular culture or language [12]. They are frequently used to quickly transmit important information such as the male or female gender (e.g., toilets info), safety hazards (e.g., health precautions) or road information (e.g., prohibitions and warnings). Although each cultural environment may promote differences in signs relevance [12,13], a basis for the use of pictograms is their universal interpretation, i.e., they should offer the same meaning regardless of language, culture or education [9,14].

Pictograms have been used to give instructions or warnings regarding health products usage [10,14]. Characteristics such as visual intricacy, concreteness, simplicity, the shape and color of the illustrations can help clarify the information conveyed or, if not well designed, misguide its assimilation [15,16,17]. As familiarity also plays a role in understanding visual aids, pictogram testing is required to determine its appropriateness [16,18]. Given most pictograms have been designed within Western societies, caution is suggested when using them in cross-cultural contexts [9,19,20].

Pharmaceutical pictograms are useful tools to reinforce both comprehension and recall of medicines-related information, attract attention and reduce misunderstandings regarding a drug treatment [13,15]. Attributes such as the design of the frames, marks expressing negation (e.g., crosses or strikethroughs), specific human body parts, and marks for pain and movement can lead to a decline in comprehension [21]. Pharmaceutical pictograms have been developed and disseminated by a few different organizations such as the Risk-benefit Assessment of Drugs-Analysis and Response (RAD-AR) Council of Japan pictograms, the United States Pharmacopeia Dispensing Information pictograms (USP) and the International Pharmaceutical Federation (FIP) pictograms [22]. The USP pictograms have been widely used in Western societies, although published studies regarding their usability and legibility in different settings revealed potential limitations for culturally diverse populations (e.g., South African) [9,10,11,23,24]. The FIP pictograms developed in June 2009 were last updated 7 February 2017, according to the website (https://www.fip.org/pictograms, accessed November 2017). This update fixed issues with the language and added Turk and Malayan, which suggest a greater potential to suit multi-cultural societies.

In Portugal, the legibility of USP pictograms was studied by Soares (2012) [25] using the overall Lisbon population. Patients’ ability to understand a set of 15 pictograms was measured according to the International Standards Organization (ISO) 3864, which considers as legible the icons presenting over 67% of correct results. Only 10 pictograms were able to achieve the legibility threshold, thus suggesting limitations in USP understanding by the Portuguese population. This was particularly relevant with low literacy and foreign communities, those justifying the development of pictograms [25]. Despite the economic recession, the influx of foreign populations has been constant in mainland Portugal. For instance, the Hindu community has been growing in Lisbon with 6160 emigrants who were born in India (before 2010), as well as from other countries such as Mozambique, Pakistan and Bangladesh [26]. Cultural minorities, who are not well versed in Portuguese, often face communication issues with treatment adherence. The use of pictograms by community pharmacy practitioners may contribute to improving adherence if the pictograms are comprehensible by all patients.

### 1.2. Study Objectives

The aim of this study was to investigate if pharmaceutical pictograms, specifically United States Pharmacopeia (USP) and the International Pharmaceutical Federation (FIP), were understandable by a Hindu-based population living abroad thus defining a feasible form of pharmaceutical communication with culturally diverse populations in Portugal. Besides the cultural sensitivity of both USP and FIP, pictograms design and other characteristics which may contribute to an enhanced meaning discernment were also investigated.

## 2. Materials and Methods

The present study followed a cross-sectional design, using a survey approach.

### 2.1. Study Participants

This study was conducted with a convenience sample of 50 Hindu individuals living in Lisbon and Tagus Valley regions of Portugal. These individuals were selected from two different Hindu temples, the Radha Krishna Temple and the Shiv Temple in Lisbon, between March and August 2017, by direct invitation from the field researcher. The inclusion criteria considered people aged over 18 years, from both genders, with different levels of education and income. Individuals who lived in India, Pakistan and/or Bangladesh (major Hindu nationalities living in Portugal) for less than five consecutive years, declaring to be unable to read Portuguese, or presenting any limitations that might prevent them from interpreting the pictograms, were excluded from the study. The selected participants responded to the questionnaire, after voluntarily signing the informed consent. From all approached and able to participate, a drop out of 18 participants was registered before achieving the 50 participants quota. The study followed all ethical research principles, particularly concerning participants’ full anonymity and data confidentiality, having received ethical approval by the Faculty of Pharmacy Ethical Committee, as well as with respect for the principles stated in the current Portuguese law of personal data protection.

### 2.2. Questionnaire

The research questionnaire consisted of three sections. The first one comprised participants’ socio-demographic data i.e., age, gender, place of birth and citizenship, level of schooling and its location, time living in Portugal, household income, employment status, and healthcare-related variables. While most of these variables were evaluated through closed multiple-choice questions, a Likert-scale was used for participants’ self-assessment of their perceived Portuguese proficiency, running from 0 (null aptitude) to 10 (native speaker). This variable was dichotomized in poor and good self-perceived Portuguese fluency, respectively ≤5 and >5 points, defining subsample B and subsample A, respectively.

The next section comprised 30 pictograms, 15 selected from the USP set and 15 from the FIP offline non-USA MEPS set. Both sets were obtained from the official websites, accessed in January 2017 (respectively, https://www.usp.org/download-pictograms and https://www.fip.org/www/?page=meps_pict_download_eu). The 15 USP pictograms were those used in previously published papers [24,25,27], 7 reported to be difficult to interpret and 8 more often correctly interpreted. The 15 FIP pictograms were those that conveyed an equal or similar meaning to the selected USP ones. It was checked if the pictograms comprised the graphics features of more than one illustration, non-affirmative marks (e.g., prohibition), and information such as warnings and precautions, besides directions. The pictograms were randomly sequenced in the questionnaire.

Each pictogram was followed by 3 descriptions, one correct and two incorrect options, written in plain European Portuguese. The pictograms correct option was obtained from the direct translation of each USP pictogram label. To develop the two incorrect options, a pilot study was conducted interviewing face-to-face 5 individual members of the Hindu community who speak and write both Portuguese and Hindi fluently. Each pictogram was shown and their interpretations noted. If their interpretation matched the correct label, two other possible options were requested, making sure those were incorrect in wording and/or meaning. If not matching the original description only one alternative interpretation was requested. This procedure produced two incorrect, but credible options, within cultural sensitivity. An informal consensus on the most suitable wrong options to use in the study was reached by the research team, which included two members of cultural minorities living in Lisbon, one from the Hindu community. The pilot study also confirmed the ease of use and completeness of the questionnaire for the members of the community.

Participants filling-in the questionnaire were asked to mark the option they considered to be correctly describing each pictogram. Each correct answer was scored with 3 points, while an incorrect answer would 1 point. A standardized final score between 1 and 3 points was obtained for all questionnaires. In the last section participants were asked about previous experiences with pictograms and to give feedback on the pictograms relevance as a patient information tool. Although this was a self-administered questionnaire, a field researcher was always present during questionnaire completion to answer any participants’ voluntary doubts.

### 2.3. Data Analysis

The analysis started by detailing pictograms classification according to the three previous graphical features, i.e., being composed of either single or multiple image, the presence or absence of any negation mark (e.g., cross or strike) and disclosing directions (e.g., how to take or apply the medication) or relaying other medication information (e.g., contraindications or side-effects). The analysis included the whole set of 30 pictograms of the USP and FIP subsets (15 + 15) i.e., no paired comparisons were intended, although equivalent pictograms from both sets were chosen.

Questionnaire data were analyzed using the IBM SPSS software, version 24. The statistics performed included descriptive results, Students’ *t*-test, Persons’ Chi-Square, non-parametric ANOVA tests and Pearson linear correlations. A confidence level equivalent to *p* < 0.05 was used in all tests.

## 3. Results

### 3.1. Socio-Demographic Data

The sample comprised mostly males (62%), with an age range between 23 and 63 years of age. Thirty-six of them declared having an Indian passport (72%), while the rest declared being citizens from Pakistan, Bangladesh or Mozambique, the last being included after confirming their main language and culture was Hindu and having lived for at least five consecutive years in that country. Overall, 44% of the respondents have lived in Portugal for up to five years, 26% between 5 and 20 years and 30% lived there for more than 20 years. Almost half (46%) of the participants had more than 12 years of education, with 66% completing their education in India, 12% studied in Portugal and the remainder studied in Pakistan, Bangladesh or Mozambique. Table 1 presents participants’ education, self-perceived Portuguese (PT) proficiency and time spent in Portugal across gender and citizenship.

Twenty-two (44%) participants rated their Portuguese fluency as 5 or below, while 28 (56%) rated their fluency as 6 or above. Hence, participants were divided into two subsamples: A speakers (*n* = 28) and B speakers (*n* = 22). The amount of time the respondents have lived in Portugal and their education level were positively associated with their self-perceived Portuguese fluency (respectively, Chi^2^ = 6.445, *p* = 0.04 and Chi^2^ = 5.547, *p* = 0.019). There was no significant association with the location where the participants acquired their Portuguese language skills, nor associations with other background variables, such as the reported household income (68% under 1000€ per month), employment status (all declared to have a job), and the healthcare provider, e.g., choosing a community pharmacist when afflicted by a minor ailment (72%) and having access to a general practice (GP) physician (64%). If presenting an ill-health condition, 52% of the participants said they would also seek traditional Hindu medical care. Only one participant admitted having a chronic condition and 39 (78%) reported taking medicines less than once a month.

### 3.2. Pictograms Data

The percentage of correct answers obtained for each individual pictogram are displayed in Table 2. Participants’ average score was 1.83 (σ = 0.34), ranging from 1.27 (the lowest score) to 2.67 (the highest). The most frequently correctly interpreted pictograms were #27, correctly interpreted by 70% of the participants, and #15 by 66% both from the UPS set. The worst interpreted pictograms were #9 (USP), with 45 (90%) participants missing the correct label and #18 (FIP) with 41 (82%) participants missing the correct label option.

No significant linear correlation was found between participants’ age and the total score. A significant negative correlation existed between the total score and the time spent outside Portugal (*r* = −0.584, *p* < 0.001) corroborated by the positive correlation for the total score and the time lived in Portugal (*r* = 0.385, *p* = 0.006). No significant differences were found between; male and female participants, the level or place of schooling, or income and employment status. A statistically significant difference was found between those having good Portuguese fluency (A participants) and poor fluency (B participants) (*t* = −3.008, *p* = 0.004). Only one participant acknowledged having had previous contact with pictograms. Thirty-eight (76%) participants considered them to be helpful for correctly understanding treatment plans.

The average USP and FIP pictograms scores were, respectively, 1.92 (σ = 0.37) and 1.74 (σ = 0.37). The USP set had a statistically significantly higher score (*t* = −3.40, *p* = 0.001) compared to the FIP. Testing the self-reported language ability (subsamples A and B) against the USP and FIP average scores confirmed differences within both pictorial sets (respectively, *t* = −2.98, *p* = 0.004 and *t* = −2.53, *p* = 0.01). The poorer Portuguese speaking participants (subsample B) showed average scores for USP of 1.75 (σ = 0.36) and for FIP of 1.59 (σ = 0.33), a difference that was significantly lower (*t* = −2.27, *p* = 0.03). Several other variables (e.g., pictograms relevance, household income, minor ailments behavior, and having a GP) were tested against participants’ average scores within all sample and subsamples and no significant associations were found.

### 3.3. Pictogram Design Data

Mean scores and standard deviations were calculated according to pictograms dichotomous graphical classifications. These were compared using Students’ *t*-test for the entire sample as well as subsamples A and B. The results are presented in Table 3.

There were 12 (40%) pictograms consisting of a single image and 18 (60%) with multiple images, the latter achieving a significantly higher total score (All: *t* = −3.91, *p* = 0.001; A: *t* = −3.09, *p* = 0.005; B: *t* = −2.62, *p* = 0.016). Thirteen (43%) pictograms had negation marks, with only a significantly higher mean interpretation score found between A participants and the entire sample (A: *t* = −3.35, *p* = 0.002) for multiple images. Sixty percent of pictograms had medication directions, while 40% had other information. As with non-affirmative signs, only the subsample A had significantly better interpretation of other information (A: *t* = −2.42, *p* = 0.022).

## 4. Discussion

Pharmaceutical pictograms are widely accepted as an important resource that provide patients with information regarding their drug therapies and believed to meaningfully contribute to safer and more effective medication use. The present study addressed the comprehension of well-known pharmaceutical pictograms by a population that does not necessarily share the cultural and linguistic background of the native population, thus requiring additional resources for effective communication.

In the present study, participants’ self-reported Portuguese fluency was found to be associated with their schooling, as well as with the time spent in Portugal, but not related to the country of formal education. On the other hand, their pictogram comprehension was significantly related to the time participants have lived in and out of Portugal. In this sense, participants’ Portuguese literacy might have been developed from an informal daily usage of the language, which suggests potential language limitations regarding less frequent and more specific contexts, such as being ill and using medication. The present study population could be an adequate means to study the usefulness of pharmaceutical pictograms.

### 4.1. Pictograms Comprehension

This study found only one pictogram (#27) out of 30 that could be immediately used in pharmacy practice, according to the ISO-3864 legibility criteria, i.e., using the 67% correct interpretation cut-off. This was lower than expected, given previous results from participants living in Portugal [25]. One cause might be the interpretation issues with reading Portuguese when choosing from the pictogram 3 label options. In fact, there was a clear association between the self-reported Portuguese fluency and average scores: the poor language proficiency group always scored worse. This confirms the common belief that effective communication issues resulting from language barriers, which frequently emerge within culturally diverse populations, may not be overcome without the effort to explain pictograms. These signs on their own might not be enough to guarantee appropriate patient information and the expected medication usage. Practitioners should keep in mind that pictograms comprehension was also independent of variables such as the frequency of using community pharmacies (for solving minor health ailments) or being in contact with a GP. Having more or less interaction with healthcare providers does not guarantee better or worse understanding of pharmaceutical pictograms, assuming the absence of translators in community pharmacies. The Hindu cultural minority, as many other minorities living in Portugal, will not necessarily be better informed just by using pictograms and hoping they will do their job. Moreover, no associations were found between different comprehension scores and appreciation of pictograms relevance, which were considered helpful to understand medication regimens. Thus, pharmaceutical pictograms are an important tool in patient counselling, although their full success requires further attention regarding the actual level of comprehension achieved by a certain population.

### 4.2. SP and FIP Comparison

FIP MEPS pictograms are a set of illustrations issued in 2009, more than one decade after the USP set released in 1997. FIP, a world-wide organization, released a pictogram software update on 7 February 2017 that fixed some language issues and included Turkish and Malayan as languages. Even if developers warn of cultural sensitivity issues, it was expected the pictograms would relay information more precisely. However, this was not confirmed: the average total comprehension score obtained with USP pictograms was significantly higher than the correspondent FIP result. This is also true when the individuals rated their Portuguese fluency as poor. This indicates that USP pictograms could be better suited to the Hindu population living in Lisbon than the FIP set, knowing pharmacists can access both freely.

### 4.3. Pictograms Design

The present study findings were not always in line with previous studies, acknowledging different research settings. The use of single images did not seem to be preferable to multiple images [17,20]. One possible explanation may be the use of several sequential frames helping the participant to better infer the meaning of subsequent images. Negatively marked pictograms were more easily interpreted, which also differs from previously published literature [18,21]. However, interpreting negative marks well (as well as medication-related information content) was achieved by those who considered themselves fluent in Portuguese. This could be a cultural feature from this sample, where these pictograms may resemble other common signs of caution to which Hindus are more sensitive. Finally, pictograms illustrating medication directions may convey more information than pictograms with warnings or precautions thus increasing complexity leading to diminished understanding among the sampled population. All these results are in line with previous findings mentioning that culture-specific and education level-specific pictograms may be essential for the effective communication of health information [28].

### 4.4. Study Limitations

Some resistance to full participation was found during the fieldwork. Bulky questionnaires with a high number of pages because of the room need for the pictures seemed to discouraged participants from completing them. As mentioned earlier, 18 selected participants dropped out before achieving the 50 participants quota, even with support from the field researcher where requested. During the pre-test, participants took an average of 11 min to complete the questionnaire but during data gathering people often took longer, mainly due to poorer understanding of the questions or to external interferences (e.g., others waiting). This apparent reluctance in completing the survey may also result from the infrequent contact due to disbelief in pictograms by community pharmacies. Using an interview approach instead of a self-administered questionnaire may have improved participation, although impact on findings is not possible to assess.

More importantly, participants showed some difficulties in reading and understanding the written information, including the wording of the options for each pictogram. While assuring a more genuine background (i.e., Portuguese is the dominant language in healthcare provision), it was not possible to control for the effects of functional cognitive abilities and literacy. No structured and independent assessment of Portuguese speaking proficiency was conducted which possibly contributed to less accurate reading of the options per pictogram and answering. This was minimized by translating questions to Hindu only when necessary, avoiding introducing undue variation in survey administration and additional response bias.

Extending the present results to all Hindu communities in Lisbon or Portugal, or other culturally diverse sub-populations, should be done with care since no representativeness or external validation was achieved in this study, resulting from anticipated time constraints. Finally, no qualitative approach was taken to investigate the reasons underlying such diverse interpretation on paired pictograms (e.g., #1 and #9 or #7 and #16), which adds further caution if the present findings are to be directly used in practice, even within the Hindu community.

## 5. Conclusions

Pictograms are potentially a good way to pass on treatment directions and precautions, in particular to culturally challenging populations, such as the Hindu Community in Portugal. Nevertheless, this study indicated that pictograms may fail their mission. Thus, it is recommended that prior to generalized usage, pictograms are tested with local populations. If local refinements are not possible, usage warnings should be issued by the responsible health authorities, alerting professionals to use them with attention.

Further field studies with pharmaceutical pictograms in Portuguese community pharmacies are needed to improve the validation and usefulness of this tool. Pictograms do not replace pharmacist–patient communication, but they cannot be ignored as an information resource in Portuguese pharmacy practice.

## Figures and Tables

**Table 1:** Participants’ demographics (*n* = 50) including variables associated with Portuguese fluency.

		Education (Years)		Self-Perc. PT Proficiency		Time Living in PT (Years)		
		≤9	>9	≤5	>5	≤5	≤20	>20
Gender	Male	15	16	15	16	15	8	8
	Female	12	7	7	12	7	5	7
	Total	27	23	22	28	22	13	15
Nationality	Portuguese	11	11	6	16	2	5	15
	Hindu	11	10	11	10	14	7	0
	Pakistani	5	0	4	1	4	1	0
	Bangladesh	0	2	1	1	2	0	0
	Total	27	23	22	28	22	13	15

**Table 2:** Pictograms used, meaning and number of correct answers per pictogram (*n* = 50).

Pictogram Id	Images	Pictogram Meaning	Correct Answers Counts (%)
#1 (FIP)	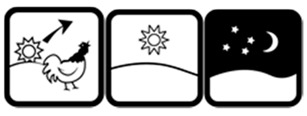	Take this medicine in the morning, afternoon and at night	19 (38%)
#2 (USP)	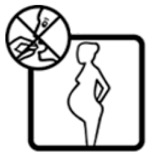	Do not take this medicine if pregnant	24 (48%)
#3 (FIP)	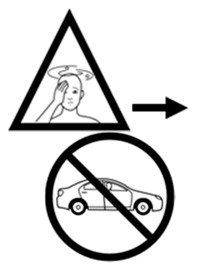	If this medicine makes you dizzy, do not drive	25 (50%)
#4 (FIP)	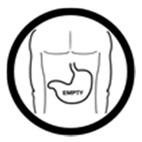	Take this medicine with an empty stomach	19 (38%)
#5 (USP)	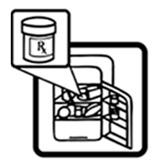	Store this medicine in the fridge	24 (48%)
#6 (USP)	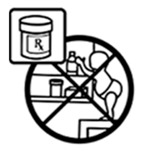	Keep this medicine out of the reach of children	32 (64%)
#7 (FIP)	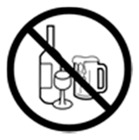	Do not drink alcoholic beverages during treatment with this medicine	15 (30%)
#8 (USP)	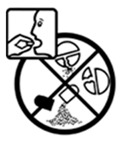	Do not break the tablets nor open the capsules	20 (40%)
#9 (USP)	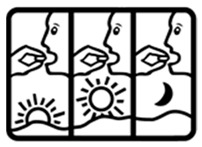	Take this medicine 3 times per day	5 (10%)
#10 (FIP)	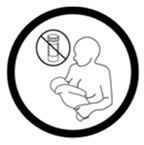	Do not take this medicine if breastfeeding	17 (34%)
#11 (USP)	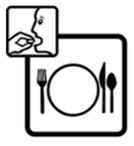	Take this medicine with meals	20 (40%)
#12 (FIP)	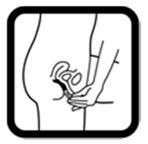	Insert the medicine in the vagina	23 (46%)
#13 (USP)	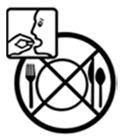	Do not take this medicine with meals	26 (52%)
#14 (FIP)	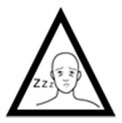	This medicine can cause sleepiness	18 (36%)
#15 (USP)	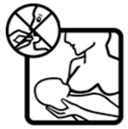	Do not take this medicine if breastfeeding	33 (66%)
#16 (USP)	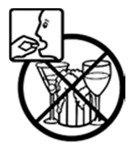	Do not drink alcoholic beverages during treatment with this medicine	23 (46%)
#17 (USP)	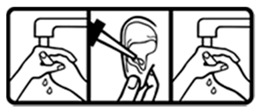	Wash your hands before and after applying this medicine on the ear	24 (48%)
#18 (FIP)	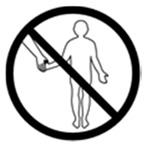	Keep this medicine out of the reach of children	9 (18%)
#19 (FIP)	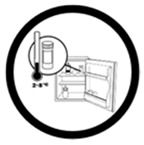	Store this medicine in the fridge	18 (36%)
#20 (FIP)	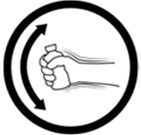	Shake this medicine before using	26 (52%)
#21 (FIP)	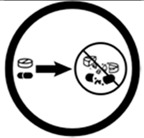	Do not break the tablets nor open the capsules	26 (52%)
#22 (FIP)	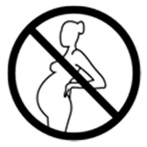	Do not take this medicine if pregnant	14 (28%)
#23 (FIP)	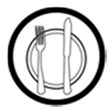	Take this medicine with meals	12 (24%)
#24 (USP)	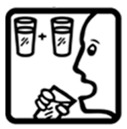	Drink this medicine with an extra glass of water	12 (24%)
#25 (USP)	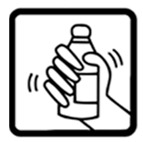	Shake this medicine before using	22 (44%)
#26 (FIP)	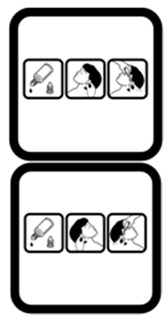	Apply one drop of this medicine on the left and on the right ears	18 (36%)
#27 (USP)	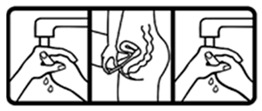	Wash your hands before and after applying this medicine on the vagina	35 (70%)
#28 (USP)	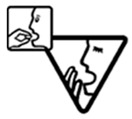	This medicine can cause sleepiness	26 (52%)
#29 (USP)	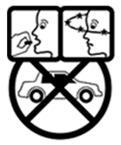	If this medicine makes you dizzy, do not drive	17 (34%)
#30 (FIP)	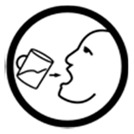	Take this medicine with water	17 (34%)

USP—United States Pharmacopeia; FIP—International Pharmacy Federation.

**Table 3:** Means and standard deviation according to pictograms design.

		Entire Sample		Subsample A		Subsample B	
		Average	σ	Average	σ	Average	σ
Images	1	1.68	0.43	1.79	0.46	1.55	0.36
	>1	1.92	0.35	2.05	0.29	1.75	0.34
Negation marks	Present	1.87	0.45	2.06	0.39	1.62	0.39
	Absent	1.80	0.32	1.86	0.32	1.72	0.31
Text	Directions	1.80	0.34	1.88	0.35	1.71	0.31
	Other info	1.86	0.43	2.04	0.37	1.62	0.39
